# Thank you to the *Journal of the Medical Library Association* reviewers in 2023

**DOI:** 10.5195/jmla.2024.1995

**Published:** 2024-04-01

**Authors:** Jill T. Boruff, Michelle Kraft, Alexander J. Carroll

**Affiliations:** 1 jill.boruff@mcgill.ca, Co-Lead Editor, *Journal of the Medical Library Association*, Associate Librarian, Schulich Library of Physical Sciences, Life Sciences, and Engineering, McGill University, Montreal, QC, Canada; 2 kraftm@ccf.org, Co-Lead Editor, *Journal of the Medical Library Association*, Medical Library Director, Cleveland Clinic Foundation, Cleveland, OH, United States; 3 alexander.j.carroll@vanderbilt.edu, Associate Director, Stevenson Science and Engineering Library, Vanderbilt University, Nashville, TN, United States

## Abstract

We sincerely thank the peer reviewers in 2023 who helped evaluate and improve the quality of work published in the *Journal of the Medical Library Association* (*JMLA*).

## PEER REVIEWERS WHO SERVED IN 2023

Ellen M. Aaronson

Rebecca Abbott

Rebecca Abromitis

Nancy Adams

Melissa Adler

Nelia Afonso

Katherine G. Akers

Karen S Alcorn

Alison M Aldrich

Nicole Allen

Kristine M. Alpi

Alice Anderson

Patricia Francis Anderson

Nicola Andrews

Pamela Andrews

Catherine Arnott Smith

Nell Aronoff

Marie T. Ascher

Erinn Aspinall

Toluwase Victor Asubiaro

Bert Avau

Ana Patricia Ayala

Lynda Ayiku

Esmaeil Bahalkeh

Sharon Bailey

Paul Bain

Michael Eliot Bales

Darra Ballance

Donna Baluchi

Mary Barnett

Jill Barr-Walker

Joan Bartlett

Kelsa Bartley

Janen Batten

Robert D. Beckett

Skye Bickett

Lindsay Blake

Catherine Boden

Roxanne Bogucka

Susan Bolton

Sarah Bonato

Ángel Borrego

C. Trenton Boyd

Alexandria Leigh Brackett

Wichor Bramer

Emily Brennan

Alison Brettle

Riley James Brian

Tara Julie Brigham

Stacy Brody

Stewart Brower

David Brown

Heather L. Brown

Janis Brown

Amelia Brunskill

John Bullion

Christopher Sean Burns

Jason Burton

Sarah Cantrell

Nicole Capdarest-Arest

Rebecca Carlson

Linda Carlson

Sarah Lynore Carnes

Tallie Casucci

Robin E. Champieux

Deborah H. Charbonneau

Amy Chatfield

Katherine Chew

Marina Chilov

Frances Chu

Natalie Clairoux

Janet Clapton

Martina Clarke

Heather Coates

Keith Cogdill

Marisa Conte

Kristen Cooper

Jeremy Cullis

John W. Cyrus

Jill Daby

Nicole Dalmer

Prudence Dalrymple

Raechel Anne Damarell

Jennifer Davis

Phil Davis

Sandy De Groote

Ariel Deardorff

Basia Delawska-Elliott

Frances A. Delwiche

Michelle Demetres

Liz Dennett

Robin Desmeules

Kerry Dhakal

Mario Enrique Diaz Barrera

Deborah Divis

Daniel Dollar

Marshall Dozier

Elizabeth Jane Dyer

Erin R.B. Eldermire

Marina Englesakis

Mette Brandt Eriksen

Julia Marie Esparza

Nina Exner

Kelly Farrah

Robin Featherstone

Lisa Federer

Bethany Figg

Jensen Fisher

Mary Gracy Flaherty

David Flynn

Barbara Folb

Elizabeth Frakes

Francesca Frati

Jacqueline Freeman

Gary Freiburger

Suzanne Fricke

Rachel Gammons

Heather Ganshorn

Gina Genova

Donna Gibson

Julie Gilbert

Kayce Gill

Shalu Gillum

Emily Ginier

Julie Glanville

Emily Glenn

Abigail Goben

Alexandra Gomes

Kelsey Grabeel

Adelia Bush Grabowsky

Vera Granikov

Charles Greenberg

Alyssa Grimshaw

Holly Grossetta Nardini

Catherine Tess Grynoch

Jean Gudenas

Karen Elizabeth Gutzman

Gale Hannigan

Conor Hanrahan

Eileen G. Harrington

Eli Harriss

Jonathan Hartmann

Dana Haugh

Andrea Hayes

Brian Haynes

Melissa Helwig

Margaret Henderson

Dean Hendrix

Ivan Herrera-Peco

Andy Hickner

Lisa Janicke Hinchliffe

Rebecca Hines

Rachel J. Hinrichs

Elizabeth Hinton

Toni Hoberecht

Kristi Holmes

Carmen Howard

Allison M. Howard

Carol L. Howe

Shanda L Hunt

Joe Hunt

Julia Alexandra Hunter

Natalie Hutchinson

Jennifer E. Isenor

Carrie Iwema

Rebecca Jacob

Janice Jaguszewski

LaTeesa James

Sarah T. Jewell

Phill Jo

Emily M Johnson-Barlow

Emily P. Jones

Soohyung Joo

Claire Joseph

Kellie Kaneshiro

Molly Kass-Kaufman

David Kaunelis

Jill R. Kavanaugh

Christiana Keinath

Julia Kelly

Erin Kerby

Jessica Kilham

Sujin Kim

Michele Klein Fedyshin

Amy C. Knehans

Helge Knüttel

Rachel Amelia Santose Koenig

Jonathan Koffel

Peter Kokol

Taneya Koonce

Jessica A. Koos

Julie Kosteniuk

Michelle Kraft

Chana Kraus-Friedberg

Janice Yu Chen Kung

Laura Kuo

Elizabeth Laera

Heather Laferriere

Lukasz Lagojda

Erica Lake

Jessica Lange

Mariana Lapidus

Fred Willie Zametkin LaPolla

Janna C. Lawrence

Emily Lawson

Mê-Linh Lê

Leila Ledbetter

Mikyoung Lee

Cedric Lefebvre

Noah Lenstra

Paul Levay

Xin Li

Brenda M. Linares

Janice Linton

Anne M. Linton

Ayaba Logan

Elizabeth R. Lorbeer

Diane L. Lorenzetti

Cuncun Lu

Ya-Ling Lu

Lili Luo

Aileen Lynch

Jennifer Lyon

Kayley Lyons

Mark MacEachern

Kendra Macomber

Keith C. Mages

Lauren Maggio

Derek Hunter Marshall

Heather J. Martin

Clément Massonnaud

Lisa Mastin

Alexa Mayo

Sean M McConachie

Lisa McGuire

Sandra McKeown

Jill McTavish

Ra Mehrab

Elizabeth Mengel

Misa Mi

Helena Mihaljevic

Jolene M. Miller

Christian I.J. Minter

Diana Mizrachi

Richard Moniz

Kaitlin Montagano

Emrys Moreau

Jane Morgan-Daniel

Rebecca Morin

Martin Morris

Whitney Mortensen

Kavita S. Mundle

Beverly Murphy

Bethany Myers

Abdolahad Nabiolahi

Christine Neilson

Joey Nicholson

Heather Nicholson

Hannah F. Norton

Rob O'Reilly

Marilyn H. Oermann

Robin O'Hanlon

Jessica Page

Lisa Palmer

Robin MN Parker

Laure Perrier

Gerald Perry

Carol L. Perryman

Jonna Peterson

Kathleen Elizabeth Phillips

Brandon Pieczko

Caitlin Pike

JJ Pionke

Josepha W.M. Plevier

Caitlin Plovnik

Kimberly R Powell

Zahra Premji

Gurpreet K. Rana

Marcia Elizabeth Rapchak

Melissa A. Ratajeski

Kevin Read

Jason B. Reed

Robyn Reed

Emily Reeve

Sophie M. Regalado

Melissa L. Rethlefsen

Rebecca Reznik-Zellen

Amy Lynn Riegelman

Kristin Rogers

Morwenna Rogers

Guillermo Armando Ronda-Pupo

Megan Rosenbloom

Amanda Ross-White

Chia Rostami

Elizabeth Roth

Stephanie Roth

Pamela Royle

Ellen Rubenstein

Ameed Saabneh

José Antonio Salvador-Oliván

Margaret Sampson

Sonia Santana Arroyo

Megan Sapp Nelson

Cathy Sarli

Kate Saylor

Frank Sayre

Lou Ann Scarton

Nancy Schaefer

Hannah Schilperoort

Cynthia Marie Schmidt

Jan W. Schoones

Carolyn Schubert

Stephanie J. Schulte

Amanda Schwartz

April J. Schweikhard

Jung Mi Scoulas

Amanda Scull

Barbara Sen

Anne Seymour

Roberta Shanman

Robert M. Shapiro

Salimah Shariff

Claire Sharifi

Chen Su-may Sheih

Tracy C. Shields

Jean Shipman

Shannon Sibbald

John Siegel

Mary Simons

Anita Slack

Judy Smith

Daniela Solomon

Jean Song

Melanie Sorsby

Judy Spak

Angela Spencer

Stuart Spore

R. Grant Steen

Madeline Sterling

Gregg A. Stevens

Julia Stumpff

Elizabeth Suelzer

Amy Marie Suiter

Alisa Surkis

Anthea Sutton

Stephanie M. Swanberg

Natalie Tagge

Maria Tan

Anneliese Taylor

Nedelina Tchangalova

Michele Tennant

Carol Tenopir

Nicole Theis-Mahon

Joanna Thielen

Holly Thompson

Jessica Thorlakson

Stephanie Tomlinson

Mary J. Tooey

J. Stephen Town

Whitney Ann Townsend

Ana Ugaz

Christine Urquhart

Saeideh Valizadeh-Haghi

Marcy Vana

Kathryn Vanderboll

Emily Vardell

Andreas Vårheim

Matt Vassar

Sarah May Visintini

Siw Waffenschmidt

Stacey Wahl

Graham Walton

Amanda Wanner

Jennifer Watling Neal

Erin Watson

Aidy Weeks

Roland Bernard Welmaker

Ju Wen

Charles Wessel

Terrie Wheeler

Rachel Whitney

Gloria Willson

Christina L Wissinger

Hannah Wood

Kath Wright

Andrea Wright

Lin Wu

Joe Wu

Lauren Yaeger

Michelle Yee

Andromeda Yelton

Semhar Yohannes

Lauren M. Young

Yan Zhang

Shirley Zhao

Carolyn Ziegler

Tyler Moses

Alicia Zuniga

